# Protective effect of tea against lead and cadmium-induced oxidative stress—a review

**DOI:** 10.1007/s10534-018-0153-z

**Published:** 2018-10-13

**Authors:** Anna Winiarska-Mieczan

**Affiliations:** Department of Bromatology and Food Physiology, University of Life Sciences in Lublin, Akademicka 13, 20-950 Lublin, Poland

**Keywords:** Tea, Antioxidants, Protective effect, Oxidative stress, Cadmium, Lead

## Abstract

Exposure to Cd and Pb reduces the activity of antioxidant enzymes, which points to a decrease in the antioxidant potential of the body as a result of supplying factors which enhance cellular oxidation processes. Man is exposed to the effects of toxic metals because they are present in the environment, including in food. Since no effective ways to reduce the concentrations of Cd an Pb in food exist, studies are undertaken to develop methods of reducing their toxic effect on the body through chelating these metals using nutrients (which reduces their absorption by tissues) or increasing the oxidative capacity of the body (which decreases the possibility of inducing oxidative damage to internal organs). Studies performed on laboratory animals have shown that the use of tea infusions fulfil both functions.

## Introduction

The presence of toxic metals in food products has become a global problem. The most significant source of toxic metals for man is food of plant origin, and in particular cereals (EFSA 2012a, b), mostly due to the fact that they are the basis of nourishment throughout the world and are consumed most abundantly. Although, according to reference literature, the content of Cd and Pb in food normally does not exceed standard levels, due to the fact that these metals are capable of accumulating in tissues and have a long half-life (Winiarska-Mieczan 2014), their regular supply, even in small amounts, is dangerous. In 2012 EFSA reduced the tolerable intake level for Cd and Pb. The TWI (Tolerable Weekly Intake) for Cd was determined at the level of 2.5 μg/kg of body weight/week (0.36 μg/kg of body weight/day) (EFSA 2012a), whereas the BMDL (Benchmark Dose Lower Confidence Limit) for Pb was: BMDL01 0.5 μg/kg of body weight/day in children and BMDL01—1.5 μg/kg of body weight/day and BMDL10 0.63 μg/kg of body weight/day in adults (EFSA 2012b).

The studies point to three main reasons for toxicity of metals: (1) the ability to react directly with proteins which results from an affinity between metals and thiol, histidine and carboxyl groups and leads to attachment of metal ions to active sites of enzymes, structural elements of cells and proteins involved in cell transport (Rubino 2015); (2) replacement of elements necessary for metabolism, e.g. calcium in bones or iron in erythrocytes with metals, which leads to damage and changes in their structure and metabolism (Puerto-Parejo et al. 2017; Jaishankar et al. 2014; Cailliatte et al. 2009); (3) participation of metals in enhancing the production of reactive forms of oxygen and modification of the activity of the antioxidant system (Mao et al. 2018; Tandon et al. 2003).

No effective methods for reducing the concentration of Cd and Pb in food exist, therefore man is constantly exposed to the intake of these metals. However, studies are being undertaken to develop methods of reducing the toxic effect of Cd and Pb on the organism through chelating these metals using nutrients (which reduces their absorption by tissues) or increasing the oxidative capacity of the body (which decreases the possibility of inducing oxidative damage to internal organs). So far, for instance, vitamin E, vitamin C, rutin and curcumin have been found to have a positive effect (Al-Attar 2011; Mirani et al. 2012; Tarasub et al. 2012). From a practical nutritional point of view it is important to examine food products containing significant amounts of antioxidant components in order to use them in a daily diet to prevent the hazardous effect of toxic metals on the human body. In available literature positive effects have been reported, among other things, for garlic, honey, rosemary and green tea (Abdel-Moneim and Ghafeer 2007; El Kader et al. 2012; Hamed et al. 2010; Padalko et al. 2012). Tea, as the most popular drink in the world apart from water, deserves particular attention (Hicks 2009). Tea contains a number of substances with an antioxidant effect such as, for example, tannic acid (Savolainen 1992), catechins (Zaveri 2006) and quercetin (Chen et al. 2009). This paper analyzes the results of surveys referring to the protective effects of tea and antioxidants it contains on organisms exposed to Pb and Cd.

## Pro-oxidant effect of Cd and Pb

Disorders of homeostasis leading to increased stationary concentrations of reactive forms of oxygen are referred to as oxidative stress. Oxidative stress induced by heavy metals can reduce the capacity of the antioxidant defence system, though lead and cadmium do not directly participate in producing reactive forms of oxygen. However, the organisms of experimental animals and people, who in their working environment were exposed to metals, showed a decrease in the resources of antioxidant vitamins (Adonaylo and Oteiza 1999; Skoczyńska 1997; Stohs and Bagchi 1995) and in the activity of endogenous antioxidant enzymes (Tandon et al. 2003) and endogenous non-enzymatic antioxidants (Mao et al. 2018). Cadmium and lead indirectly contribute to oxidative stress, secondarily leading to increased peroxidation of lipids, damage to nucleic acids, alterations in the expressions of genes and apoptosis processes, inhibiting the activity of antioxidant proteins by binding them to sulfhydryl groups and inhibiting calcium homeostasis (Czeczot et al. 2009; Fowler et al. 2004; Ercal et al. 2001; Stohs and Bagchi 1995). Their participation in creating free oxygen radicals and their derivatives is not only manifested in a disturbed flow of electrons in the respiratory chain, but also in the release of transition metals involved in the Fenton and Haber–Weiss reactions—mainly Fe(II) and Cu(I) from the sites in which they occur in cells (e.g. ferritine, ceruloplasmin, proteins containing iron–sulphur clusters in the respiratory chain, heme proteins and other) (Stohs and Bagchi 1995; Waisberg et al. 2003; Valko et al. 2006). A higher amount of reactive forms of oxygen in cells exposed to the effect of toxic metals can also be a result of the deteriorated function of antioxidant mechanisms. This is mostly due to the decreased concentration of reduced glutathione (GSH) in cells, total pool of protein-bound –SH groups and changes in the activity of antioxidant enzymes (Nemmiche 2017).

Reactive oxygen species include one- (superoxide anion radical O_2_^·−^), two- (hydrogen peroxide H_2_O_2_) and three-electron (hydroxyl radical HO·) products of oxygen reduction and singlet oxygen, as well as ozone (O_3_) or organic free radicals (Wu et al. 2011). The presence of reactive oxygen species in cells can lead to oxidative damage to antioxidant enzymes. For instance, these include catalase the activity of which is inhibited directly by O_2_^·−^ (Kono and Fridovich 1982) or superoxide dismutase which in turn is inactivated by peroxynitrite (MacMillan-Crow et al. 1998). A secondary effect of changes in the structure of these enzymes can be an increase in the pool of H_2_O_2_ and O_2_^·−^ in cells, which was demonstrated in studies on plant cells (Schützendübel and Polle 2002). Reactive oxygen species react with polyunsaturated fatty acids in cell membranes, which initiates the process of lipid peroxidation resulting in the modification of proteins, changes in the electrochemical gradient, which in turn gives rise to a loss of their integrity and to irreversible damage (Lambert and Elias 2010). If an unpaired electron is present, such molecules are characterized by high reactivity since they aim at pairing the electrons by accepting or giving them away. Given the current status of knowledge the mechanism of Cd an Pb toxicity involves inducing oxidative stress in cells, which results in the first place in peroxidative damage to cell membranes (Lambert and Elias 2010).

Cytotoxicity of heavy metals is limited by antioxidant enzymes such as superoxide dismutase (SOD), catalase (CAT) and glutathione peroxidase (GPX), converting oxygen species into molecular oxygen and water (Pourahmad and O’Brien 2000; Valko et al. 2006) and by non-enzymatic antioxidants occurring in cells, and in particular glutathione (GSH) (Pereira et al. 2013; Valko et al. 2006).

## Endogenous antioxidant mechanisms

In the course of evolution all living organisms developed a number of enzymatic and non-enzymatic defence mechanisms tasked with maintaining reactive oxygen species at a low level safe to cells. Their main task is neutralizing free radicals, inhibiting free radical chain reactions and protecting the cell against their toxic effect (Liczmański 1988). The most important defence mechanisms of the body are connected with the antioxidant effect of SOD, CAT, GPX and GST (glutathione transferase). Short-term exposure to toxic metals enhances the activity of SOD, CAT, GPX and glutathione reductase, which points to the activation of defence mechanisms and adaptive response of the cells. When the exposure lasts longer, their activity in cells clearly decreases, which is a result of Mn, Cu and/or Zn ions being displaced from the active site of MnSOD in the case of CuZnSOD or Fe ions from the heme group of catalase or Se ions from glutathione peroxidase (Dalle-Donne et al. 2008; Patra et al. 2011; Adi et al. 2016).

SOD is an enzyme occurring in cytosol (cytoplasmic matrix) and mitochondria. With regard to the type of metal present in the active site, three SOD classes can be distinguished: copper-zinc (Cu,Zn-SOD), manganese (Mn-SOD) and ferrous (Fe-SOD). This enzyme catalyzes the reaction of dismutation of superoxide anion radical (O_2_^·−^) producing hydrogen peroxide (H_2_O_2_) and molecular oxygen (O_2_) as a result of reduction and oxidation of metal ions forming a part of active sites of SOD. In peroxisomes, hydrogen peroxide is subject to further enzymatic dismutation to water and oxygen involving CAT. This compound is also a substrate for peroxidases which reduce it to water, oxidizing different compounds occurring in cells in reduced forms. In plants this is a role of ascorbate peroxidase occurring in chloroplasts and in cytoplasm and using ascorbate as a specific donor of electrons. This enzyme interacts with monodehydroascorbate reductase and dehydroascorbate reductase which regenerate ascorbate at the cost of oxidizing glutathione as well as with glutathione reductase which recreates the reduced glutathione in the reaction of oxidation of NADPH (Ighodaro and Akinloyeb 2017).

CAT is located in peroxisomes and is involved in inactivating H_2_O_2_ that is a side product of oxidation of fatty acids (Fujiwara et al. 2000). It is Fe-protoporphyrin composed of four monomers with heme group as the active site. CAT shows double activity: catalase and peroxidase activity. For high concentrations of hydrogen peroxide the basic function of the enzyme is its participation in a two-stage H_2_O_2_ disproportionation reaction. At the first stage, as a result of heme iron being oxidized by hydrogen peroxide, porphyrin cation radical is formed, whereas at the second stage the transitional reaction product is reduced to iron with oxidation state 3 + by another molecule of hydrogen peroxide, as a result of which molecular oxygen and water are formed (Wołonciej et al. 2016).

GPX is a selenoenzyme acting as catalyst for the reduction of hydrogen peroxide by GSH. The reaction produces an oxidized form of glutathione (glutathione disulfide). GPX plays a significant role as a system of defence against reactive oxygen species; it protects both the cells and the extracellular area (Li et al. 2000). It participates in the first and second line of defence against free radicals. Glutathione peroxidase directs the attack of hydrogen peroxide onto glutathione preventing its participation in the Fenton reaction and thus protecting the thiol groups of proteins and reducing organic peroxides to alcohols (Li et al. 2000). Eight forms of glutathione peroxidases were described—they occur in most internal organs and also in erythrocytes, cytosol, and mitochondrion and in the cell nucleus (Wołonciej et al. 2016).

GSH is a particulate sulfhydryl compound—an important non-enzymatic component of the antioxidant system of cells. In the cells it is not only an internal redox buffer, a direct “sweeper” of reactive oxygen species, but also a co-substrate in the reactions of inactivating reactive oxygen species and detoxicating the xenobiotics catalyzed by GSH-dependent enzymes (Dalle-Donne et al. 2008). Because GSH participates in direct binding of prooxidant metal ions, its total level in cells is reduced, which contributes to increased oxidative stress. The –SH glutathione group is much more accessible to oxygen than the thiol groups if enzymes. Therefore, GSH can secure biologically active proteins (Dalle-Donne et al. 2008).

## The antioxidant properties of tea

Studies carried out by many authors have shown a high antioxidant potential of extracts of different types of tea (Awoniyi et al. 2011; Gawlik and Czajka 2007; Karori et al. 2007; Toschi et al. 2000). Consumption of teas intensifies the antioxidant capacity of the body—it contributes to an increased activity of basic antioxidant enzymes such as: glutathione reductase, glutathione peroxidase, catalase, glutathione S-transferase and quinone reductase. It is mostly observed in the liver, small intestine and lungs (Michalak-Majewska 2011). It was demonstrated that the antioxidant potential in blood plasma after drinking green tea is increased by 34%, while after drinking black tea the increase is 29% (Serafini et al. 1996). In vitro tests involving human erythrocyte preparations showed that green tea was characterized by higher antioxidant activity than white and black tea, which was evaluated based on the level of α- and γ-tocopherol in the analyzed cells (Gawlik and Czajka 2007). In turn, studies of a system simulating the process of oxidation occurring in the human body indicated that green and black tea infusions inhibited linoleic acid peroxidation very strongly and in a similar way (Wołosiak et al. 2008). Korir et al. (2014) presented similar results in their experiments involving mice. The results of clinical trials showed that green tea (single dose of 5 g extract/150 ml water) delays LDL oxidation (Ohmori et al. 2005). The LDL oxidation delay phase, measured by the amount of conjugated dienes, reaches the maximum 2 h after drinking tea. The protective effect of green tea on LDL is a result of an increase in the concentration of respective catechins in blood plasma, because the maximum concentration of catechins occurs 2 h after drinking tea, which coincides with the maximum of the LDL oxidation delay phase. Drinking green tea also increases the antioxidant activity of human blood plasma, depending on the dose (Sung et al. 2000). The overall antioxidant capacity after drinking 2 cups of tea (1 cup = 2.5 g leaves/150 ml water) compared to water is 7% higher in 60 min and 6.2% higher in 120 min. Increasing the dose to 3 cups of tea increases the antioxidant activity of plasma by 12% in 60 min, whereas this activity remains unchanged for at least up to 120 min after drinking tea. In turn, Serafini et al. (1996) demonstrated that drinking 300 ml of green tea increases the antioxidant capacity of blood plasma by 40% in 30 min. However, even 80 min after consumption, this value reaches the baseline. Studies carried out by Ambrożewicz et al. (2010) revealed that black tea has an even stronger effect than green tea on the antioxidant system of the human umbilical vein endothelial cells under oxidative stress induced by tert-butyl hydroperoxide. In addition, infusions from both types of tea prevented oxidative modification of lipids and proteins to a significant extent.

It is believed that the antioxidant properties of tea result from the high content of polyphenols such as catechins (Zaveri 2006), including epigallocatechin-3-gallate (EGCG) in green tea (Kim et al. 2014), quercetin (Chen et al. 2009), theaflavins and thearubigins in black tea (Gramza et al. 2005) and tannic acid (Savolainen 1992). Total polyphenols account for 25–35% of the dry matter of tea leaves (Bharadwaz and Bhattacharjee 2012). The highest total content of polyphenols (presented as an equivalent of tannic acid) is found in white tea (2668 mg per 1000 ml), followed by green tea (2363 mg per 1000 ml), black tea (1220 mg per 1000 ml) and red tea (996 mg per 1000 ml) (Winiarska-Mieczan 2015).

**Fig. 1:**
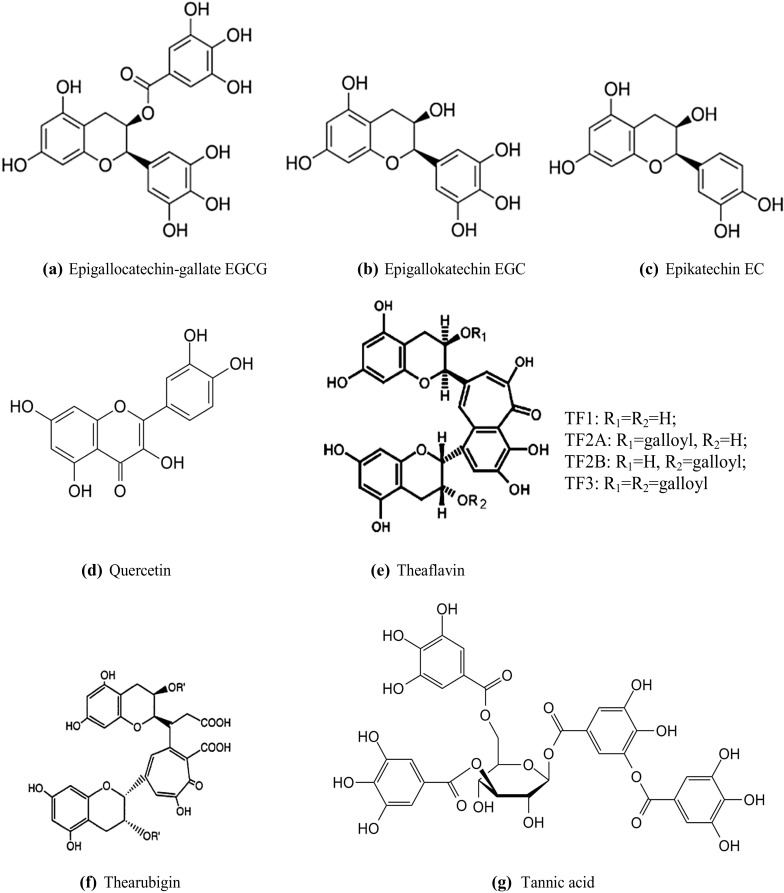
Chemical structure of some major polyphenols in tea

Catechins, believed to be the most important antioxidants in tea, were studied very thoroughly. They are colourless water-soluble substances which make the infusion taste bitter and have an astringent effect (Bharadwaz and Bhattacharjee 2012). Catechins account for 8–15% of the dry matter of tea leaves (Choung et al. 2013). Green tea, that is not subject to fermentation, contains more catechins than fermented teas such as oolong (partial fermentation) or black and Pu-erh teas (complete fermentation) (Toschi et al. 2000). During fermentation of O_2_ oxidoreductase, involving polyphenol oxidase, monophenol monooxygenase and o-diphenol, about 75% of catechins contained in leaves are subject to enzymatic oxidation (Donejko et al. 2013; Kusano et al. 2015; Kuhnert 2010). The content and type of catechins in green tea depends on the variety of tea, climate and growing conditions (Toschi et al. 2000). Also, the conditions in which tea infusions are made have an impact on the content of catechins in the infusion. The most effective infusions are made at 80 °C for 40 min (Choung et al. 2013). In turn, infusions made at a temperature of about 100 °C have catechins content lower by even 15% (Gramza et al. 2005). Studies showed that in 60 min after drinking green tea, human blood plasma contains 3 times more catechins than after drinking black tea (Leenen et al. 2000). The predominant type of catechins found in tea infusions is EGCG (Fig. 1a), (Karori et al. 2007). In addition, epigallocatechin (EGC; Fig. 1b), epicatechin (EC; Fig. 1c) and epicatechin gallate (ECG) have a large share. The classic antioxidant effect of catechins is based on their reducing properties, including direct inactivation of reactive oxygen and nitrogen species and decreasing the production of reactive oxygen species, as well as an indirect effect being the regeneration of other antioxidants such as α-tocopherol or β-carotene and chelating transitional metals (Kim et al. 2014; Abib et al. 2011). Studies involving rats as subjects revealed that EGCG had higher bioactivity than EGC and EC (Rietveld and Wiseman 2003). The condition for high antioxidant activity of EGCE is the presence of eight –OH groups (Gramza et al. 2005). Young tea leaves contain EGCG > EGC > ECG > EC, mature leaves EGC > EGCG > ECG > EC, whereas old ones EGC > EGCG > EC > ECG (Karori et al. 2007). Studies by Xu et al. (2004) concerning the antioxidant activity of catechins present in green tea noted a considerable increase in the antioxidant potential of the blood plasma of rats after administration of an oral dose of a mixture of catechins in the amount of 4000 mg/kg of body weight. The maximum antioxidant activity was observed in the 40th min after administration of the mixture, which coincides with the maximum concentration of catechins in blood plasma in time.

Quercetin (Fig. 1d) is a phytocompound from a group of flavonoids of plant origin, demonstrating a wide range of properties, for instance antioxidant, anti-inflammatory and immunomodulating properties. Antioxidant properties of quercetin result from its ability to “sweep” reactive oxygen species, inhibiting the activity of enzymes participating in the creation of reactive oxygen species, such as oxidases, enzymes, with substrates such as, for instance, purine derivatives (e.g. kinases, ATPases, adenylyl cyclase, reverse transcriptase, DNA and RNA polymerases, ribonuclease) and enzymes using NADPH as a co-enzyme (e.g. aldose reductase, lactate dehydrogenase, nitric oxide synthase, glutathione reductase). Quercetin also shows the ability to modulate the activity of enzymes involved in antioxidant processes, e.g. SOD and GST (Dolinoy et al. 2006; Kobylińska and Janas 2015; El-Sayed and Rizk 2009). The antioxidant capacity of quercetin is closely connected with its chemical structure (possibility to give away an electron or hydrogen atom), thanks to which it can neutralize singlet oxygen (^1^O_2_), superoxide anion radical (O_2_^·−^), hydroxyl radical (OH), peroxyl radicals (LOO), nitrogen oxide (NO) and peroxynitrite (ONOO–) (Amorati et al. 2017; Amić et al. 2017; Dolinoy et al. 2006). The antioxidant activity of quercetin is also manifested in its ability to capture alkoxy radicals by giving away one OH electron, and stabilizing the resulting alkoxy radicals thanks to an aromatic ring (Dolinoy et al. 2006).

Black teas are dominated by theaflavins (Fig. 1e) and thearubigins (Fig. 1f), because during the process of fermentation of tea leaves catechins are condensed into larger polyphenol molecules (Wang et al. 2016). Theaflavins are formed at the first stage of fermentation. At the following stages oxidation products are transformed into thearubigins (Bailey et al. 1994; Kusano et al. 2015). So far at least 28 derivatives of theaflavin have been recognized. Theaflavin (TF1), theaflavin-3-gallate (TF2A), theaflavin-3′-gallate (TF2B) and theaflavin-3,3′-digallate (TF3) are predominant in black tea. Studies of the antioxidant activity of teas clearly indicate that the activity of theaflavins and that of catechins is comparable. Theaflavin-3,3′-digallate in black tea shows antioxidant activity similar to that of EGCG in green tea extract (Karori et al. 2007; Wu et al. 2011; Yoshino et al. 1994). Studies showed effectiveness of theaflavins in reducing oxidation of human LDL as TF3 > ECG ≥ EGCG ≥ TF2B ≥ TF2A > TF1 ≥ EC > EGC (Leung et al. 2001). Thearubigins, accounting for about 60% of polyphenolic compounds in black tea extract, are strongly oxidized polymeric polyphenolic fractions (Kuhnert 2010; Menet et al. 2004). Tests involving rats showed that both theaflavin and thearubigin are effective inhibitors of peroxidation in liver cells (Yoshino et al. 1994).

Tannins present in tea are a product of polyphenolic oxidation (Chung et al. 1998). According to Sánchez-Moreno et al. (2000), tannins are better antioxidants than other commonly used antioxidants such as vitamin C and E. Tannic acid (Fig. 1g) has very strong antioxidant properties. According to Pulido et al. (2000) tannic acid is characterised by a higher antioxidant capacity than other polyphenols and this capacity is not lower than that of BHA, BHT and α-Tocopherol (Gülcin et al. 2010). Tannic acid in in vitro conditions inhibits almost 98% of peroxidation of lipids at the concentration of 15 μg/ml, while standard antioxidants (e.g. BHA and α-Tocopherol) have similar results at the concentration of 45 μg/ml. Tannic acid also demonstrates a comparable capability of chelating metals (Gülcin et al. 2010). Tannic acid is absorbed from the alimentary tract in mice and sheep, it is found in blood plasma (Zhu et al. 1992) and can have a chelating effect on toxic metals present in blood and internal organs. The highest content of tannic acid in 1000 ml of tea infusion (prepared in distilled water at a temperature of 90° C for 5 min) occurs in white and green tea (more than 110 mg), followed by black tea (94 mg) and red tea (77 mg) (Winiarska-Mieczan 2015).

It was demonstrated that a particularly high antioxidant capacity of green and white tea results from the fact that these teas do not differ significantly in terms of the content of total polyphenols, catechins and antioxidant activity (Karori et al. 2007).

## Protective effect of tea on organisms exposed to Cd and Pb

### Antioxidant effect

**Fig. 2:**
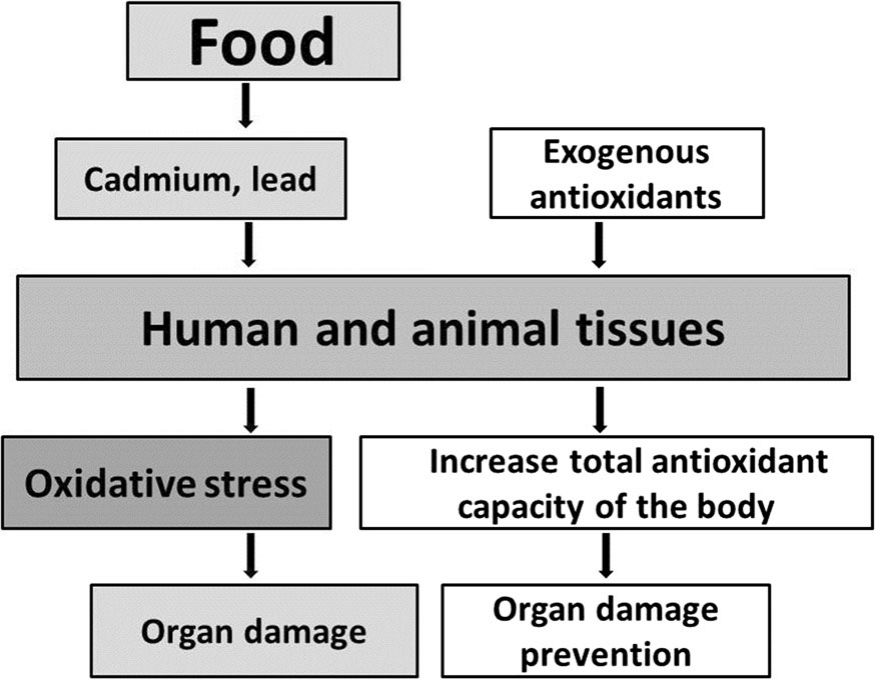
Efficiency of antioxidants mechanisms resulting from the supply of exogenous antioxidants

Exposure to Cd and Pb reduces the activity of antioxidant enzymes, which points to a decrease in the antioxidant potential of the body as a result of supplying agents enhancing cellular oxidation (Ramesh and Satakopan 2010; Wei and Meng 2011). The increase in the activity of SOD, CAT, GST and GPX and an increase in the content of GSH in the organs of animals (rats, chickens) receiving tea solutions (Tables 1, 2, 3), observed in numerous studies, indicates increased efficiency of antioxidant mechanisms resulting from the supply of exogenous antioxidants, which facilitates maintaining a balance in redox reactions and prevents oxidative stress (Fig. 2). Other studies revealed that the activity of endogenous antioxidant enzymes is increased as a result of supplying antioxidants only during long-term exposure to Cd and Pb (Winiarska-Mieczan 2013), which indicates that only regular, continuous drinking of tea ensures positive results. Also, other authors using tea demonstrated that it supports antioxidant processes in the body exposed to Cd and Pb. El-Shahat et al. (2009) revealed that green tea extract administered to rats receiving Cd in the form of a water-based solution containing 0.4% CdCl_2_ reduced the degree of peroxidation of lipids in testicles, thus preventing damage. According to Khalaf et al. (2012) green tea extract administered to rats poisoned with Pb in the amount of 100 mg/kg of body weight for 15 days increased the activity of antioxidant enzymes, including SOD, in the brain. According to studies by Hamed et al. (2010), Meki et al. (2011) and Abdel-Maneim et al. (2014), after using an extract of green tea in rats poisoned with a 0.4% lead acetate solution for 6 weeks, the activity of GST, SOD and the content of GSH in the brain, blood, liver and kidneys increased in comparison to the control group (water). In the quoted studies a reduction in the degree of peroxidation of lipids was found in the analyzed organs. Studies regarding the brain are particularly worth attention because this is an organ that is critical with regard to the toxic effect of Cd and Pb. Prolonged exposure to Cd and Pb causes brain damage as a result of oxidative stress (Adonaylo and Oteiza 1999; Flora et al. 2008). This is due to its high consumption of oxygen, high content of lipids and relatively low content of antioxidant enzymes in the organ. Particularly significant alterations in the activity of antioxidant enzymes can be observed in the cerebral mitochondria, which are the main source of superoxide anion radical and hydrogen peroxide (Tian et al. 1998). The brains, lungs, hearts, livers and kidneys of rats poisoned simultaneously with Cd and Pb (7 mg Cd and 50 mg Pb/kg feed), drinking green, black, red and white tea, showed an increased activity of SOD, CAT and GPx and an increased level of GSH compared to the control group (Winiarska-Mieczan 2015). The effectiveness of teas increased along with the duration of the experiment (6 vs. 12 weeks). Also, Cd injected in rats with a water-based green tea solution (Hamden et al. 2009; Kumar et al. 2010a, b) significantly increased the activity of exogenous antioxidants in the blood and liver.

**Table 1:** Effect of tea on an organism exposed to Pb

Tea	Protective effect	Pb dose and design	Animals	Target sites	References
Green tea	↑ TAC; ↑ RGSH; ↑ SOD; ↓ DNA fragmentation	100 mg of lead acetate/kg bw by gastric tube for 1 month; green tea in drinking water (5 g/l) orally for 1 month	Albino male rats	Brain	Khalaf et al. (2012)
Green tea	↑ GST; ↑ RGSH; ↑ SOD; ↑ TAC; ↓ LPO; ↓ Pb	0.4% aqueous solution of lead acetate orally for 6 weeks; green tea in distilled water (15 g/l) orally for 6 weeks	Rats	Brain, blood	Hamed et al. (2010)
Green tea	↑ SOD; ↓ LPO; ↑ GST; ↓ Pb accumulation	0.4% aqueous solution of lead acetate orally for 6 weeks; green tea in drinking water (15 g/l) orally for 6 weeks	Male rats	Liver, kidney, brain	Meki et al. (2011)
Green tea	↓ LPO; ↑ SOD; ↑ GST; ↑ GSH; ↓ LPO; ↓ Pb accumulation; ↑ urea in blood; ↓ creatinine in blood	0.4% aqueous solution of lead acetate orally for 6 weeks; green tea in drinking water (15 g/l) orally for 6 weeks or mixture of 0.4% lead acetate + green tea solution (15 g/l)	Male Sprague–Dawley rats	Kidney	Abdel-Maneim et al. (2014)
Green tea	↓ ALT; ↓ AST; ↓ TC; ↓ LDL; ↓ TG; ↑ HDL	500 mg lead acetate/kg diet daily for five weeks; green tea extract (882 mg/kg bw/day) orally injected for five weeks	Male albino rats	Blood	El-Ziney et al. (2017)
Green tea	↑ total protein; ↑ albumin; ↑ SOD; ↑ GST; ↓ AST; ↓ ALT; ↓ ALP; ↓ Pb concentration in liver	0.4% aqueous solution of lead acetate orally for 30 days; 6.6% green tea extract orally for 30 days	Male Wistar rats	Liver, blood	Hamadouche et al. (2014)
Green tea	↓ MDA; ↑ GSH; ↑ SOD; ↑ CAT; ↓ Pb accumulation;	200 mg Pb (as lead acetate)/kg basal diet for 42 days; 1 g green tea based probiotic/kg basal diet for 42 days	Ross broiler chicks	Liver, blood	Yosef et al. (2012)
Green tea	↓ Pb accumulation; ↓ LPO; ↑ GSH; ↑ GST; ↑ SOD	Mixture of 1.5 green tea extract and 0.4% lead acetate/l distilled water for 6 weeks	Male Sprague–Dawley rats	Testes	Essa et al. (2009)
Green tea	↑ total protein; ↑ albumin; ↓ AST; ↓ ALT; ↓ ALP; ↓ Pb accumulation; ↑ SOD; ↑ GST	0.4% aqueous solution of lead acetate orally for 8 weeks; 1.5% green tea extract in drinking water for 8 weeks	Male Sprague–Dawley rats	Liver	Mehana et al. (2012)
Green tea	↓ LPO; ↑ CAT; ↑ SOD; ↑ GPX	0.4% aqueous solution of lead acetate orally for 4 weeks; 6.6% green tea extract orally for 4 weeks	Male Wistar rats	Kidneys	Hamadouche et al. (2015)
Green tea	↓ MDA; ↑ SOD; ↑ GSH	1 g/l drinking water lead acetate/day orally for 8 weeks; 1.5% green tea extract orally for 8 weeks	Male Sprague–Dawley rats	Testes	El-Beltagy et al. (2015)

↑ increased concentration or activity compared to Pb group, ↓ decreased or inhibited concentration or activity compared to Pb group, *RGSH* reduced glutathione, *GSH* glutathione, *SOD* superoxide dismutase, *CAT* catalase, *ALP* alkaline phosphatase, *LPO* lipid peroxidation, *TAC* total antioxidant capacity, *GPX* glutathione peroxidase, *GST* glutathione S-transferase, *MDA* malondialdehyde, *LDL* low density lipoprotein, *HDL* high-density lipoprotein, *TG* triglicerides, *TC* total cholesterol, *ALT* alanine aminotransferase, *AST* aspartate aminotransferase, *bw* body weight

**Table 2:** Effect of tea on an organism exposed to Cd

Tea	Protective effect compared to Cd treated animals	Cd dose and design	Animals	Target sites	References
Green tea	↑ sugar; ↑ protein; ↑ ALP; ↓ ACP; ↓ ALT; ↓ AST	10 mg dose of cadmium chloride/kg bw by oral route; green tea in drinking water (20 or 40 mg/kg bw) orally for 15 or 30 days	Male Wistar rats	Liver	Singh et al. (2013a)
Green tea	↑ Hb; ↑ RBC; ↑ PCV; ↑ TLC; ↑ MCV	10 mg dose of cadmium chloride/kg bw by oral route; green tea in drinking water (20 or 40 mg/kg bw) orally for 15 or 30 days	Male Wistar rats	Blood	Singh et al. (2013b)
Green tea	↓ LDL; ↓ GGT; ↓ ACP; ↓ ALP; ↓ bilirubin; ↑ SOD; ↑ CAT; ↑ GPX; ↓ TBARs	20 µmoles of cadmium/kg bw/every 3 days for 6 months by injection; 5% green tea extract in drinking water for 12 h daily during 6 months	Male Wistar rats	Liver, blood	Hamden et al. (2009)
Green tea	↓ GSH; ↓ TBARs; ↑ CAT; ↑ GPX	1.25 mg cadmium chloride/kg bw by injection; 1.5% green tea extract in drinking water for 45 days	Male Wistar rats	Liver	Kumar et al. (2010a)
Green tea	↓ SGOT; ↓ SGPT; ↓ LDH; ↓ GGT	1.25 mg cadmium chloride/kg bw by injection; 1.5% green tea extract in drinking water for 45 days	Male Wistar rats	Blood	Kumar et al. (2010b)
Green tea	↑ LH; ↑ FSH	first 400 mg cadmium chloride/l distilled water orally by 21 days and 7 or 14 mg/l green tea solvent through 21 consecutive days	Female Wistar rats	Blood	Mahmood et al. (2015)
Black tea	↓ TG; ↓ LDL; ↑ HDL; ↑ liver structure	1 mg cadmium chloride/kg bw for 21 days; 2.5% of aqueous solution of black tea extract orally for 21 days	Male Wistar rats	Liver, blood	Mantur et al. (2014)
Kombucha tea	↓ AST; ↓ ALT; ↓ ALP; ↑ TAC; ↑ SOD; ↑ CAT; ↑ GSH; ↓ TBARs; ↓ MDA	3.5 mg cadmium chloride/kg bw by injection (single dose); kombucha tea ferment during 2 weeks before cadmium chloride injection and 4 weeks after injection	Male albino rats	Liver, kidney, blood	Ibrahim (2013)
Green tea	↓ MDA; ↑ SOD; ↑ GSH	4 g/l drinking water cadmium chloride/day orally for 8 weeks; 1.5% green tea extract orally for 8 weeks	Male Sprague–Dawley rats	Testes	El-Beltagy et al. (2015)

↑ increased concentration or activity compared to Cd group, ↓ decreased or inhibited concentration or activity compared to Cd group, *SOD* superoxide dismutase, *CAT* catalase, *TAC* total antioxidant capacity, *GPX* glutathione peroxidase, *ALP* alkaline phosphatase, *ACP* acid phosphatase, *GSH* glutathione, *TG* triglicerides, *HDL* high-density lipoprotein, *LDL* low-density lipoprotein, *TBARs* thiobarbituric acid reactive substances, *GGT* gamma-glutamyl transferase, *MDA* malondialdehyde, *LH* luteinizing hormone, *FSH* follicle-stimulating hormone, *SGOT* glutamate oxaloacetate transaminase, *SGPT* glutamate pyruvate transaminase, *LDH* lactate dehydrogenase, *GGT* γ-glutamyl transferase, *Hb* haemoglobin, *RBC* red blood cells, *PCV* packed cell volume, *TLC* total leucocyte count, *MCV* mean corpuscular volume, *ALT* alanine aminotransferase, *AST* aspartate aminotransferase, *bw* body weight

**Table 3:** Effect of tea on an organism exposed simultaneously to Cd and Pb

Tea	Protective effect compared to Pb treated animals	Pb and Cd dose and design	Animals	Target sites	References
Green, black, red and white	↓ Cd and Pb accumulation	7 mg Cd (as cadmium chloride) and 50 mg Pb (as lead acetate)/kg of feed for 12 weeks; infusions of teas as a sole source of drink for 12 weeks	Male Wistar rats	Femur bone, blood	Tomaszewska et al. (2016)
Green, black, red and white	↓ Cd accumulation; ↓ Pb accumulation in liver; ↑ SOD; ↑ CAT; ↑ GSH; ↑ GPX	7 mg Cd (as cadmium chloride) and 50 mg Pb (as lead acetate)/kg of feed for 6 and 12 weeks; infusions of teas as a sole source of drink for 6 and 12 weeks	Male Wistar rats	Lungs, brain, heart, liver, kidneys	Winiarska-Mieczan (2015)

↑ increased concentration or activity compared to Cd/Pb group, ↓ decreased or inhibited concentration or activity compared to Cd/Pb group, *SOD* superoxide dismutase, *CAT* catalase, *GSH* glutathione, *GPX* glutathione peroxidase

Increased activity of endogenous antioxidant mechanisms in the bodies of animals poisoned with Cd and Pb was also observed when isolated antioxidants naturally occurring in tea were used (Table 4). Most studies refer to quercetin having a proven strong antioxidant effect (Dolinoy et al. 2006; Kobylińska and Janas 2015; El-Sayed and Rizk 2009). 75 mg of quercetin, administered orally to rats poisoned with 4 mg Cd/kg of body weight for 2 weeks caused an increase in the level of antioxidants in testicles such as: SOD, GPX and GSH (Bu et al. 2011). Renugadevi and Prabu (2009, 2010) presented similar results in their studies of blood, liver and kidneys of rats poisoned with 5 mg Cd/kg of body weight for 4 weeks,. The rats simultaneously received 50 mg of quercetin. In addition, these authors observed an increase in the level of vitamin C and E in the studied organs. Zargar et al. (2015) in addition checked the lipid peroxidation level (LPO), obtaining confirmation that this ratio in the brains of rats receiving quercetin injections (100 mg/kg of body weight) was reduced in comparison to the control group. Also, the administration of quercetin (50 mg/kg of body weight per day) caused an increase in the antioxidant potential in the kidneys of rats poisoned with 500 mg Pb/kg of body weight/day in the form of a water-based solution for 10 weeks, which was manifested in an increased activity of SOD, CAT, GPX and GSH/GSSG (Liu et al. 2010). Similarly, Yuan et al. (2016) found that in the livers of mice poisoned with 0.4 mg Cd/kg of body weight/day and simultaneously receiving 5–100 mg of quercetin per kg of body weight per day, the oxidation level of lipids and reactive oxygen species decreased and the total antioxidant capacity increased. In the studies by Nna et al. (2017) female rats were given 5 mg Cd (as CdCl_2_) per kg of body weight per day in different combinations: without quercetin (control group) and with 20 mg of quercetin/kg of body weight per day—6 h before, 6 h after or simultaneously with cadmium chloride. The uteruses and ovaries of these rats showed an increased antioxidant potential (increase in the activity of SOD, CAT, GPX and GSH level, decrease in MDA and H_2_O_2_ level) compared to the control group. Similar results were presented by Milton Prabu et al. (2013) who in their studies involving rats receiving simultaneously Cd (50 mg/kg bw/day) and quercetin (5 mg/kg bw/day) for 4 weeks found an increase in the level of antioxidant parameters in blood and hearts.

**Table 4:** Effect of antioxidants naturally occurring in tea on an organism exposed to Pb and/or Cd

	Protective effect compared to Pb and/or Cd treated animals	Pb and Cd dose and design	Animals	Target sites	References
Quercetin	↓ MDA; ↓ H_2_O_2_; ↑ SOD; ↑ GPX; ↑ GSH; regulation the expression of proapoptotic protein	4 mg cadmium chloride/kg bw orally daily for 2 weeks; 75 mg quercetin/kg bw orally daily for 2 weeks	Male ICR mice	Testes	Bu et al. (2011)
Quercetin	↓ AST; ↓ ALT; ↓ ALP; ↓ LDH; ↓ GGT; ↓ TBARS; ↓ hydroperoxides; ↓ protein carbonyls; ↑ vitamin C; ↑ vitamin E; ↑ SOD; ↑ CAT; ↑ GPX; ↑ GST; ↑ GSH	5 mg cadmium chloride/kg bw/day orally for 4 weeks; 50 mg quercetin/kg bw/day orally prior to the administration of Cd for 4 weeks	Male Wistar rats	Blood, liver	Renugadevi and Prabu (2009)
Quercetin	↓ LPO; ↓ ascorbic acid; ↑ GSH; ↑ CAT; ↑ SOD	2 mg/kg bw cadmium fluoride by injection for 24 or 48 h; 100 mg/kg bw quercetin by injection for 24 or 48 h	Male and female mice	Liver	Zargar et al. (2015)
Quercetin	↓ ROS; ↑ GSH/GSSG; ↑ CAT; ↑ SOD; ↑ GPX; ↑ GSH	500 mg Pb (as lead acetate)/l drinking water orally for 10 weeks; 10 mg/kg bw/day quercetin by oral gavage for 10 weeks	Male Wistar rats	Kidney	Liu et al. (2010)
Quercetin	↓ Cd and Pb accumulation; ↑ CAT; ↑ SOD; ↑ GPX; ↓ MDA; ↓ H_2_O_2_	mg/kg bw/day cadmium chloride; 20 mg/kg bw/day quercetin 6 h before or 6 h after cadmium chloride or simultaneously	Female Wistar rats	Uteri, ovaries	Nna et al. (2017)
Quercetin	↓ MDA; ↑ CAT; ↑ SOD; ↑ GPX; reduced the Cd-induced histopathological changes	1 mg Cd (as cadmium chloride)/kg bw/day by injection for 30 days; 15 mg quercetin/kg bw for 30 days	Male Sprague–Dawley rats	Brain	Unsal et al. (2015)
Quercetin	↓ TBARS; ↑ GSH; ↑ TSH; ↑ vit. C and E; ↑ CAT; ↑ SOD; ↑ GPX; ↑ GST; ↓ hydroperoxide; ↓ protein carbonyls	5 mg Cd (as cadmium chloride)/kg bw for 4 weeks; 50 mg quercetin/kg bw orally prior to the administration of cadmium for 4 weeks	Male Wistar rats	Blood, kidney	Renugadevi and Prabu (2010)
Catechin	↓ PLA; ↓ COX; ↓ TXA; ↓ PGI; ↓ TBARS	500 ppm Cd (as cadmium chloride)/l distilled water orally by 20 weeks; 2.5 or 5 g catechin/kg diet by 20 weeks	Male Sprague–Dawley rats	Blood	Choi et al. (2003)
Tannic acid	↓ Cd and Pb accumulation	50 mg Pb (as lead acetate) or 7 mg Cd (as cadmium chloride)/l distilled water orally for 6 or 12 weeks; 2% solution of tannic acid in drinking water orally alternatively every 7 days with 100 mg Pb or 14 mg Cd for 6 or 12 weeks	Male Wistar rats	Heart, lungs	Winiarska-Mieczan et al. (2013)
Tannic acid	↓ Cd accumulation; ↑ SOD after 12 weeks; ↑ CAT both after 6 and 12 weeks	7 mg Cd (as cadmium chloride) and 50 mg Pb (as lead acetate)/kg of feed for 6 or 12 weeks; tannic acid with drink (0, 0.5, 1, 1.5, 2 or 2.5% solutions) for 6 or 12 weeks	Male Wistar rats	Brain	Winiarska-Mieczan (2013)
	↓ Cd and Pb accumulation; ↑ SOD after 12 weeks; ↑ CAT both after 6 and 12 weeks	aqueous solutions of [Cd (7 or 14 mg/l distiller water) or Pb (50 or 100 mg/l distilled water)] or 2% tannic acid solution, alternatively every 7 days, for 6 or 12 weeks			
Tannic acid	in blood: ↓ Cd and Pb; in bones: ↓ Cd and Pb; ↑ cancellous parameters; ↑ articular cartilage constituents	7 mg Cd (as cadmium chloride) and 50 mg Pb (as lead acetate)/kg of feed for 6 weeks; tannic acid with drink (0, 0.5, 1, 1.5, 2 or 2.5% solutions) for 6 weeks	Male Wistar rats	Femur bone, blood	Tomaszewska et al. (2017b)
Tannic acid	in blood: ↓ Cd and Pb; in bones: ↓ Cd and Pb; ↑ weight and length; ↑ ultimate strength and max. elastic strength; ↑ articular cartilage constituents	7 mg Cd (as cadmium chloride) and 50 mg Pb (as lead acetate)/kg of feed for 12 weeks; tannic acid with drink (0, 0.5, 1, 1.5, 2 or 2.5% solutions) for 12 weeks	Male Wistar rats	Femur bone, blood	Tomaszewska et al. (2017a)
Tannic acid	↓ Pb; ↓ AST; ↓ ALT	20 mg Cd/kg/day by oral gavage for 4 weeks; tannic acid with drink (0.5, 1.0 or 2.0 mg/kg/day) for 4 weeks	Female mice	Blood, liver, kidney	Kim et al. (1998)
Tannic acid	↓ AST; ↓ ALT; ↓ ALP; ↓ cholesterol; ↑ total protein; ↑ albumin; ↑ globulin	200 ppm cadmium acetate orally by 12 weeks; 200 ppm tannic acid orally by 12 weeks	Female rats	Blood	Al-Fartosi (2010)
Tannic acid	↓ LPO; ↓ protein carbonylation; ↑ SOD; ↑ CAT; ↑ dehydrogenases	0.44 mg cadmium chloride/kg bw administered subcutaneously by 15 days; 12, 25 or 50 mg tannic acid/ml orally by 15 days	Male Wistar rats	Blood, liver, kidney	Mishra et al. (2015)
Tannic acid	↓ LPO; ↑ GSH; ↑ GST; ↑ GPX; ↑ SOD; ↑ CAT	50 mg/kg bw lead acetate intraperitoneally three times a week for two weeks; 50 mg/kg bw tanic acid orally three times a week for two weeks	Male Wistar rats	Brain	Ashafaq et al. (2016)
Polyphenols	↓ Pb; ↓ urea; ↓ creatinine; ↓ cell apoptosis; ↓ mRNA expression; ↓ ROS	500 mg/l Pb (as lead acetate) orally for 60 days; 20 or 50 mg/kg bw/day green tea polyphenols by oral gavage for 6 weeks	Male Wistar rats	Kidney	Wang et al. (2016)
Polyphenols	↓ AST; ↓ ALT; ↑ liver structure	50 mg cadmium sulfate/l drinking water orally for 30 days; 400 mg/kg bw green tea polyphenols by oral gavage for 30 days	Female albino rats	Blood, liver	Al-Gnami (2014)
Polysaccharides	↓ Pb; ↑ ALAD; ↓ ROS; ↓ MDA; ↑ GSH; ↑ SOD; ↑ CAT	0.2% lead acetate by gavage drink for 6 weeks; 50, 100 or 200 mg/kg bw/day red tea polysaccharides by gavage for 6 weeks	Male Kunming mice	Liver, kidney, blood	Li and Liu (2014)

↑ increased concentration or activity compared to Cd/Pb group, ↓ decreased or inhibited concentration or activity compared to Cd/Pb group, *SOD* superoxide dismutase, *CAT* catalase, *LPO* lipid peroxidation, *ROS* reactive oxygen species, *TAC* total antioxidant capacity, *GPX* glutathione peroxidase, *GST* glutathione S-transferase, *MDA* malondialdehyde, *GSH* detecting glutathione, *GSSG* glutathione disulfide, *PLA* plateled phospholipase A2, *COX* plateled cyclooxygenase, *TXA* thromboxane A2, *PGI* prostacyclin, *TBARS* thiobarbituric acid reactive substance, *ALAD* d-aminolevulinic acid dehydratase, *GGT* gamma glutamyl transferase, *MDA* malondialdehyde, *TSH* thyroid stimulating hormone, *ALT* alanine aminotransferase, *AST* aspartate aminotransferase, *ALP* alkaline phosphatase, *LDH* lactate dehydrogenase, *bw* body weight

In addition, studies were carried out to check the effect of other antioxidants in tea on the antioxidant potential of laboratory animals poisoned with Cd and/or Pb (Table 4). Giving 0.5, 1, 1.5, 2 or 2.5% tannic acid solution to rats receiving 7 mg Cd and 50 mg Pb/kg of feed for 6 or 12 weeks increased the activity of SOD and CAT in their brains in comparison to those which were given water to drink (Winiarska-Mieczan 2013). Similarly, Mishra et al. (2015) showed an increase in the antioxidant potential (decreased LPO, increased activity of SOD, CAT and dehydrogenases: isocitrate, alpha-ketoglutaric, succinic and pyruvate dehydrogenase) in rats receiving simultaneously 0.44 mg of cadmium chloride and water-based solutions of tannic acid containing 12, 25 or 50 mg of this substance in comparison to those receiving only CdCl_2_. Similarly, the use of tannic acid (50 mg per kg of body weight, 6 times for two weeks) in rats poisoned with lead acetate (50 mg per kg of body weight intraperitoneally) intensified the endogenous protection of the body (decreased LPO, increased GSH, GST, GPX, SOD, CAT) in the brains compared to the control group (not receiving tannic acid). Also in livers, kidneys and blood of rats poisoned with 2% solution of lead acetate for 6 weeks an increase in SOD and CAT was observed in groups receiving polysaccharides isolated from red tea in the amount of 50, 100 or 200 mg/kg of body weight (Li and Liu 2014). Wei and Meng (2011) revealed that in the ventricular myocytes of rats poisoned with Pb, the activity of antioxidant ratios after the use of EGCG was increased (increased activity of SOD and CAT, reduction in MDA, OH·, O_2_^·−^ and H_2_O_2_).

### Chelating effect

The chelating effect of polyphenols on metals is connected with the content of dihydroxyl and tri-hydroxyl groups (Khokhar and Owusu Apenten 2003). The effect of chelating is reduced absorption of Cd and Pb in tissues.

Tests were performed on rats receiving Cd (7 mg/kg of feed) and Pb (50 mg/kg of feed) in parallel with giving (green, white, black, red) tea infusions to those animals as the only drink for 12 weeks (Tomaszewska et al. 2016; Winiarska-Mieczan 2015). The level of metals was measured in the femoral bone, lungs, heart, liver and kidneys. The results obtained showed the highest effectiveness of reducing the absorption of Cd and Pb in the case of a white and green tea solution, while black tea was the least effective. The authors attribute this to the fact that white and green tea contains more polyphenols, including catechins, capable of chelating metallic elements, than in black and red tea (Karori et al. 2007). In the present authors own studies (Winiarska-Mieczan 2015), it was also observed that tea solutions efficiently reduced the degree of accumulation of Cd in tissues. Pb was less susceptible to the chelating effect of the ingredients of teas. It was found that, compared to other organs, the teas were the least effective both with regard to Cd and Pb in the lungs. This can also suggest that drinking tea will not considerably level, for example, the adverse impact of smoking tobacco that is one of the most important sources of Cd and Pb for humans and simultaneously the most important cause of lung cancer (Fowles and Dybing 2003). Polyphenols present in tea reveal strong anticarcinogenic properties (Lambert and Elias 2010), which in this case may not be fully utilized. Pb is more resistant to the chelating effect of polyphenols than Cd is since Pb demonstrates a strong affinity with thiol groups which are absent in polyphenols (Winiarska-Mieczan 2013; Aykin-Burns et al. 2005) but are present in calcium ion transporter protein (Cailliatte et al. 2009), which is the reason for the accumulation of Pb in bones replacing calcium. The studies revealed that people exposed to Pb had low levels of calcium but higher concentrations of parathormone (Anetor et al. 2005) responsible for regulating calcium and phosphates (Mudipalli 2007). According to El-Shahat et al. (2009) green tea extract effectively chelates Cd, thanks to which it does not participate in prooxidative processes in the body.

Some authors claim that catechins are the main factor behind inhibiting the absorption of Cd and Pb in rats. Studies by Abib et al. (2011) involving mitochondria isolated from the brains of rats showed that EGCG forms inseparable complexes with Cd^2+^, which prevents their absorption, whereas binding effectiveness was higher at higher pH values. Paul (2008) and Choi et al. (2003) obtained similar results. However, An et al. (2014) in their studies on human liver cells found that EGCG had a minimum chelating effect on Cd^2+^. On the other hand, those authors admitted that EGCG facilitated maintaining the redox homeostasis in the analyzed tissues.

Studies revealed that the use of polyphenols isolated from green tea (20 or 50 mg/kg of body weight/day) in rats poisoned with 500 mg Pb for 60 days resulted in a significant decrease in the level of Pb in kidneys compared to the group receiving Pb only (Wang et al. 2016). In the uteruses and ovaries of female rats receiving Cd (5 mg/kg of body weight per day) and quercetin 6 h before, 6 h after or simultaneously with the administration of Cd, the level of Cd was found to have decreased (Nna et al. 2017). However, based on data available in reference literature, apparently tannic acid is one of the major components of tea determining the preventive effect of tea in relation to Cd and Pb. Tests involving rats proved that tannic acid reduces the absorption of Cd and Pb by tissues, which is a result of the chelating properties of this acid (Winiarska-Mieczan 2013). Kim et al. (1998) demonstrated that the tissues of mice poisoned with 20 mg Cd per kg of body weight contained less Cd if the mice drank a water-based solution containing 0.5, 1.0 or 2.0 mg of tannic acid/ml. The use of a 2% tannic acid solution in rats receiving 7 mg Cd and 50 mg Pb/kg of feed or one litre of distilled water contributed to a significant decrease in the accumulation of those toxic metals in the hearts, lungs, tibia and blood compared to rats drinking water (Winiarska-Mieczan et al. 2013; Winiarska-Mieczan 2013; Tomaszewska et al. 2017a, b). Studies showed that Pb was a more resistant metal than Cd to being bound by tannic acid (Winiarska-Mieczan 2013), where the use of 0.5, 1, 1.5 or 2% tannic acid solutions in adult rats simultaneously exposed to Cd and Pb resulted in a statistically significant reduction of Cd absorption in brains by 20–25% after 6 weeks of the experiment and about 35% after 12 weeks. However, it had no significant effect on the level of Pb. This could be due to the fact that Pb reveals a considerably stronger affinity with thiol groups (Aykin-Burns et al. 2005) which do not occur in polyphenolic compounds than with hydroxyl groups present there. In addition, it was found that in rats drinking a tannic acid solution in parallel to receiving feed this substance was more effective than when the solution was administered alternately (every 7 days) with feed contaminated with Cd and Pb (Winiarska-Mieczan 2013).

## Summary

To sum up, the protective effect of teas on the body against toxic metals must be considered in the context of the summative effect of various active substances present in infusions, as it cannot be excluded that their simultaneous effect may be different from their effect if used separately and certainly their effect is summative. Thus, it is difficult to determine which antioxidant present in tea infusions has the strongest effect.
